# Comprehensive Analysis Identified ASF1B as an Independent Prognostic Factor for HBV-Infected Hepatocellular Carcinoma

**DOI:** 10.3389/fonc.2022.838845

**Published:** 2022-02-24

**Authors:** Xianmo Wang, Huawei Yi, Jiancheng Tu, Wen Fan, Jiahao Wu, Li Wang, Xiang Li, Jinrong Yan, Huali Huang, Rong Huang

**Affiliations:** ^1^ Clinical Laboratory, The First People’s Hospital of Jingzhou, The First Affiliated Hospital of Yangtze University, Jingzhou, China; ^2^ Clinical Laboratory, The Second Clinical College of Wuhan University, Wuhan, China; ^3^ Department of Neurology, The Fifth Affiliated Hospital of Guangxi Medical University, Nanning, China; ^4^ Department of Neurology, The First People’s Hospital of Nanning, Nanning, China

**Keywords:** lung cancer, HBV, ASF1B, prognosis, ASF1B overexpression

## Abstract

**Purpose:**

Hepatitis B (HBV)-infected hepatocellular carcinoma is one of the most common cancers, and it has high incidence and mortality rates worldwide. The incidence of hepatocellular carcinoma has been increasing in recent years, and existing treatment modalities do not significantly improve prognosis. Therefore, it is important to find a biomarker that can accurately predict prognosis.

**Methods:**

This study was analyzed using the The Cancer Genome Atlas (TCGA) database and validated by the International Cancer Genome Consortium (ICGC) database. The STRING database was used to construct a gene co-expression network and visualize its functional clustering using Cytoscape. A prognostic signature model was constructed to observe high and low risk with prognosis, and independent prognostic factors for HBV-infected hepatocellular carcinoma were identified by Cox regression analysis. The independent prognostic factors were then analyzed for expression and survival, and their pathway enrichment was analyzed using gene set enrichment analysis (GSEA).

**Results:**

805 differentially expressed genes (DEGs) were obtained by differential analysis. Protein–protein interaction (PPI) showed that DEGs were mostly clustered in functional modules, such as cellular matrix response, cell differentiation, and tissue development. Prognostic characterization models showed that the high-risk group was associated with poor prognosis, while Cox regression analysis identified ASF1B as the only independent prognostic factor. As verified by expression and prognosis, ASF1B was highly expressed in HBV-infected hepatocellular carcinoma and led to a poor prognosis. GSEA showed that high ASF1B expression was involved in cell cycle-related signaling pathways.

**Conclusion:**

Bioinformatic analysis identified ASF1B as an independent prognostic factor in HBV-infected hepatocellular carcinoma, and its high expression led to a poor prognosis. Furthermore, it may promote hepatocellular carcinoma progression by affecting cell cycle-related signaling pathways.

## Introduction

Liver cancer is one of the most common cancers worldwide and is the leading cause of cancer-related deaths. The incidence of liver cancer has continued to increase in recent years and has been concentrated to countries with high sociodemographic indices (1). There were more than 900,000 new cases of liver cancer in 2020, with the location of occurrence concentrated to the eastern, southeastern, and northern regions of Asia (2). In addition, there are gender and ethnic differences in the incidence of liver cancer (3). Currently, liver cancer is still very difficult to treat. Although it can be treated by surgical resection, organ transplantation, and chemotherapy, patients’ prognoses have not improved greatly in recent years (4). The prognosis prediction of hepatocellular carcinoma relies mostly on clinical phenotype and biochemical features, but their prognostic value is limited. Therefore, the search for a new biomarker to improve diagnostic accuracy and prognostic prediction is particularly important.

Many risk factors are involved in the development of hepatocellular carcinoma. Significant among these risk factors are viral infections and poor lifestyles (5). The vast majority of liver cancer cases can be attributed to chronic viral infections, with hepatitis B virus (HBV) being one of the major types (6). HBV is a small hepatophilic DNA virus that replicates by selectively infecting hepatocytes and undergoing retrotransposition (7). Upon the infection of liver tissue, HBV double-stranded DNA integrates with the human genome and promotes hepatocellular carcinoma progression through oncogenic activation or inactivation (8). The nature of this behavior is to induce genetic instability, affect tumor-associated signaling pathways, and alter the immune response, thereby disrupting normal hepatocyte programming. Chronic infection *via* the HBV virus usually leads to acute or chronic hepatitis and cirrhosis, followed by progressive evolution to hepatocellular carcinoma (9). The HBV vaccination is currently the main way to prevent HBV infection, and it has been effective in reducing the incidence of infection in endemic areas (10). However, the total number of liver cancer deaths due to HBV infection is still increasing (11). This may be because HBV may mediate genetic mutations and tumor heterogeneity in hepatocellular carcinoma tissues, resulting in a different therapeutic sensitivity to chemotherapy than in HBV-negative hepatocellular carcinoma patients (12).

In recent years, research has focused on identifying factors associated with the prognosis of HBV-infected hepatocellular carcinoma. A clinical study showed that a family history of hepatocellular carcinoma was associated with reduced overall prognostic survival (13), several miRNAs were found to predict survival in patients with hepatitis B-related hepatocellular carcinoma (5), and 17 pivotal genes were identified as potential prognostic markers for HBV-infected hepatocellular carcinoma (14). In this study, we cut through patients with liver cancer due to HBV infection to find new biomarkers associated with liver cancer prognosis.

## Data and Methods

### Data Sources

Gene expression matrix and clinical information were obtained from the TCGA database, which yielded 371 cases of liver cancer tissue samples and 50 cases of normal tissue samples following a search. The samples were divided into HBV-positive (145 cases) and HBV-negative (226 cases) groups based on whether the patients were infected with HBV. In addition, the gene expression profiles of hepatocellular carcinoma were downloaded from the International Cancer Genome Consortium (ICGC) database, which included 243 cases of hepatocellular carcinoma tissue samples and 202 cases of paraneoplastic tissues.

### Differential Analysis

The limma package in R was used to study the differential expression of mRNA. The liver cancer samples were divided into HBV infection-positive and HBV-negative groups, and genes with differential expression in the two groups were sought. |log2FC|<0.3785, P<0.05 was defined as the screening threshold for differentially expressed genes (DEGs).

### Protein-Protein Interaction Networks (PPI)

DEGs were imported into the STRING database (https://string-db.org/) to obtain gene-to-gene interaction network relationships. Cytoscape was used to visualize gene interaction network relationship maps and to perform module screening of PPI networks using the MCODE plugin. Metascape (https://metascape.org/gp/index.html#/main/step1) was used to resolve the biological pathway enrichment and gene function annotation of the interaction networks.

### Minimum Absolute Contraction and Selection Operator Analysis

Dimensionality reduction and the construction of prognostic feature models were achieved based on the least absolute shrinkage and selection operator (LASSO). LASSO regression was performed using the R package glmnet to screen potential prognostic risk features. A risk score formula was obtained from the model, which was used to calculate the risk score for each sample. All patients were divided into high- and low-risk groups based on median values. The R packages survival and survminer were used to analyze survival differences between the two groups, and the R package timeROC was used to analyze the receiver operating characteristic (ROC) analysis performed at 1, 3, and 5 years. Cox risk proportional regression analysis was used to determine the independent prognostic factors in the prognostic model.

### Expression and Survival Analysis

The Wilcoxon rank-sum test was used to analyze the gene expression distribution in the two groups of samples, and mapping was achieved with the R package ggplot2. Survival analysis was performed using the R package surv, and log rank was used to test Kaplan–Meier (KM) survival analysis and to compare the survival differences between the two groups. P < 0.05 was considered statistically significant.

### Single Gene Set Enrichment Analysis

Single gene set enrichment analysis (GSEA) was used on the TCGA dataset to identify the biological pathways between the high and low ASF1B expression groups of HBV-infected hepatocellular carcinoma. |NES|>1, NOM p-val<0.05, and FDR q-val<0.25 were defined as screening thresholds based on which the Kyoto Encyclopedia of Genes and Gene (KEGG), and Hallmark signaling pathways were obtained. The top biological processes that were altered were selected according to the normalized enrichment score (NES) ranking.

## Results

### Identification of Differentially Expressed Genes Between HBV-Positive and HBV-Negative Samples

**Figure 1:**
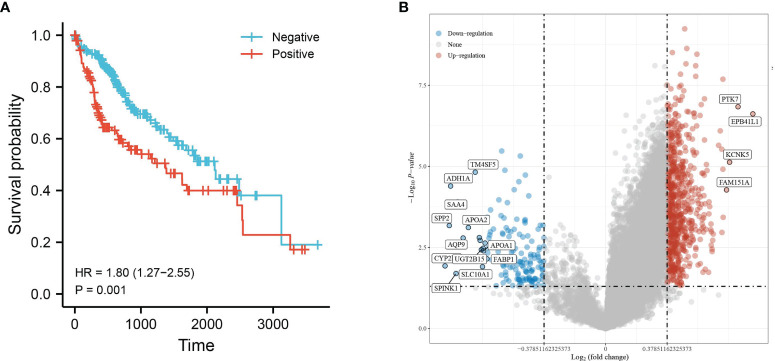
Differential gene expression analysis of HBV infection-positive and negative hepatocellular carcinomas. **(A)** KM survival curves based on groupings of HBV infections or not and **(B)** volcano plot with upregulated genes in red and downregulated genes in blue.

Hepatocellular carcinoma tissue samples from the TCGA database were divided into two groups, HBV infection positive and negative, and the clinical information was compared (**Table 1**). The prognosis of hepatocellular carcinoma associated with positive HBV infection was found to be worse by survival analysis (**Figure 1A**). Therefore, this was used for differential analysis to seek DEGs, and a total of 805 genes were found to be significantly differentially expressed. Of these, 703 were upregulated genes, and 147 were downregulated genes (**Figure 1B**).

**Table 1:** Comparison of clinical information.

characteristics	C1	C2	P_value
OS	145	226	0.002
Gender	145	226	0.962
Race	238	373	0.57
T_stage	144	225	0
N_stage	144	226	0.173
M_stage	145	226	0.857
TNM_stage	140	207	0
Grade	144	222	0.081
New_tumor_event_type	74	99	0.005

C1 is HBV-positive, C2 is HBV-negative.

### Identification of Key Functional Gene Modules

**Figure 2:**
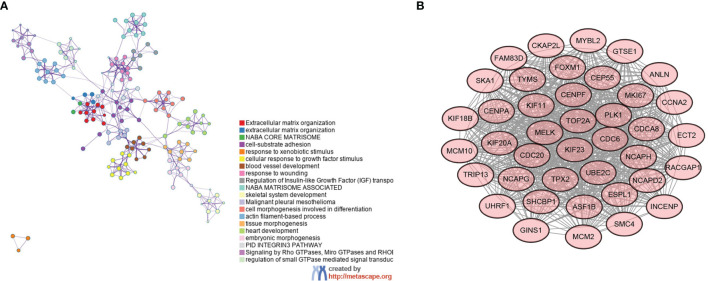
Gene interaction network diagram and key gene modules. **(A)** Functional interaction network relationship diagram of genes—different colors represent different functional clusters—and **(B)** key gene modules.

The STRING database was used to analyze the gene–gene interactions, Metascape was used to show the enrichment of gene functional terms, and Cytoscape was used to visualize this result. All DEGs have functional linkages between them, and they are classified into different modules by functional clustering (**Figure 2A**). The most representative functional terms or pathways in the top 20 functional clusters are shown in **Table 2**, and these include cell–matrix response, cell differentiation, and tissue development. The gene interaction network map was subdivided into 30 gene modules using the MCODE plugin. The module with the largest score (score: 37.231), which contains 40 nodes and 726 edges (**Figure 2B**). Each node represents a gene, and the higher the score of the module, the more critical and typical the genes are. The genes in this module were selected for subsequent analysis.

**Table 2:** Top 20 functional terms.

Category	Description	P
Reactome Gene Sets	Extracellular matrix organization	7.41E-27
GO Biological Processes	extracellular matrix organization	1.23E-26
Canonical Pathways	NABA CORE MATRISOME	3.72E-25
GO Biological Processes	cell-substrate adhesion	1.26E-23
GO Biological Processes	response to xenobiotic stimulus	2.57E-22
GO Biological Processes	cellular response to growth factor stimulus	1.02E-21
GO Biological Processes	blood vessel development	1.29E-21
GO Biological Processes	response to wounding	1.74E-20
Reactome Gene Sets	Regulation of Insulin-like Growth Factor (IGF) transport and uptake by Insulin-like Growth Factor Binding Proteins (IGFBPs)	9.55E-20
Canonical Pathways	NABA MATRISOME ASSOCIATED	4.57E-19
GO Biological Processes	skeletal system development	2.82E-18
WikiPathways	Malignant pleural mesothelioma	4.27E-18
GO Biological Processes	cell morphogenesis involved in differentiation	3.47E-16
GO Biological Processes	actin filament-based process	5.89E-16
GO Biological Processes	tissue morphogenesis	9.33E-16
GO Biological Processes	heart development	2.45E-15
GO Biological Processes	embryonic morphogenesis	2.63E-15
Canonical Pathways	PID INTEGRIN3 PATHWAY	1.74E-14
Reactome Gene Sets	Signaling by Rho GTPases, Miro GTPases and RHOBTB3	9.77E-14
GO Biological Processes	regulation of small GTPase mediated signal transduction	3.80E-13

### High-Risk Score Is Associated With Poorer Prognosis

**Figure 3:**
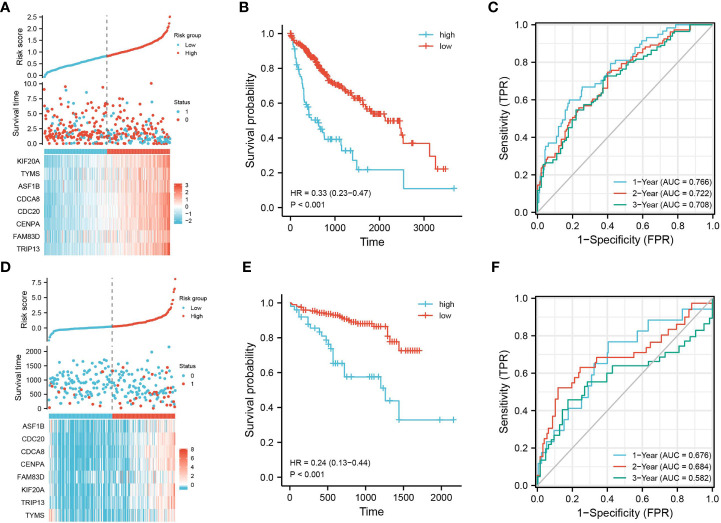
Construction of prognostic feature model. Using the TCGA dataset as the test set, **(A)** the distribution of high- and low-risk samples; **(B)** KM survival curve; **(C)** time-dependent ROC curve; using the ICGC dataset as the validation set, **(D)** distribution of high- and low-risk samples; **(E)** KM survival curve; and **(F)** time-dependent ROC curve.

The prognostic characteristic risk model was used to study the prognostic effects of 40 genes in key modules on HBV infection related liver cancer samples. The model is a riskscore formula containing multiple genes, and each gene has weight. The analysis showed that the model contained 8 genes. The risk score was calculated using Riskscore = (0.0103)*KIF20 A + (-0.0493)*TYMS + (-0.1866)*ASF1B + (0.1975)*CDCA8 + (0.0657)*CDC20 + (0.1702)*CENPA + (0.0192)*FAM83 D + (0.1364)* TRIP13, which was used for each sample. Using the TCGA dataset as the test set and the ICGC dataset as the validation set, the samples were divided into high- and low-risk groups based on the median cut-off value (cut = 1.2), and **Figure 3A** shows their distribution. The survival of different risk groups for HBV-infected hepatocellular carcinoma is shown in **Figure 3B**, with the high-risk group pointing to a worse prognosis. The predictive power of this prognostic risk model was demonstrated by ROC curves, with more accurate predictions at 1 and 3 years (**Figure 3C**). The model was thereafter validated using the ICGC dataset, and the results were consistent with the test set, showing that the high-risk group was associated with a poorer prognosis (**Figures 3D**–**F**).

### ASF1B Is an Independent Prognostic Factor for HBV-Infected Hepatocellular Carcinoma

**Figure 4:**
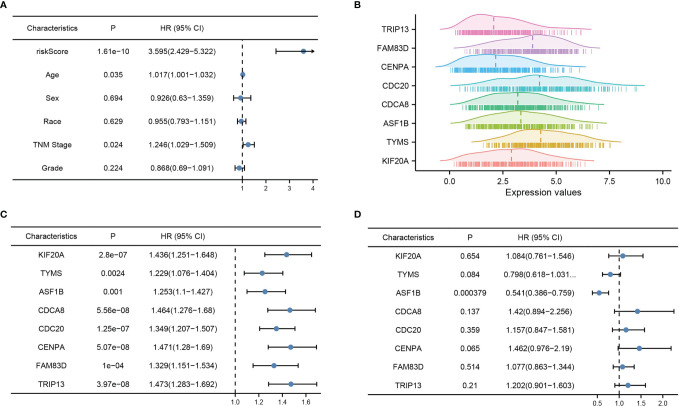
Risk model validation and independent prognostic factor analysis. **(A)** Multi-factor Cox analysis of risk score and each clinical factor, **(B)** mountain range plot of each factor in the risk score formula, **(C)** one-way Cox analysis of each factor in the risk score formula, and **(D)** multi-way Cox analysis of each factor in the risk score formula. p < 0.05 was considered statistically significant.

Multi-factor Cox regression analysis was performed using risk score values and each clinical phenotype. Among them, risk score (P = 0), age (P = 0.035), and TNM stage (P = 0.024) were significant, indicating that the above prognostic risk score model is credible (**Figure 4A**). The mountain range plot demonstrates the distribution of the eight prognostic features in the risk score formula for HBV-infected hepatocellular carcinoma, with the dashed line showing the median of each feature (**Figure 4B**). Subsequent univariate and multifactorial Cox regression analyses were performed, and ASF1B was found to be an independent prognostic factor for HBV-infected hepatocellular carcinoma (**Figures 4C, D**).

### High ASF1B Expression Is Associated With a Poor Prognosis in HBV-Infected Hepatocellular Carcinoma

**Figure 5:**
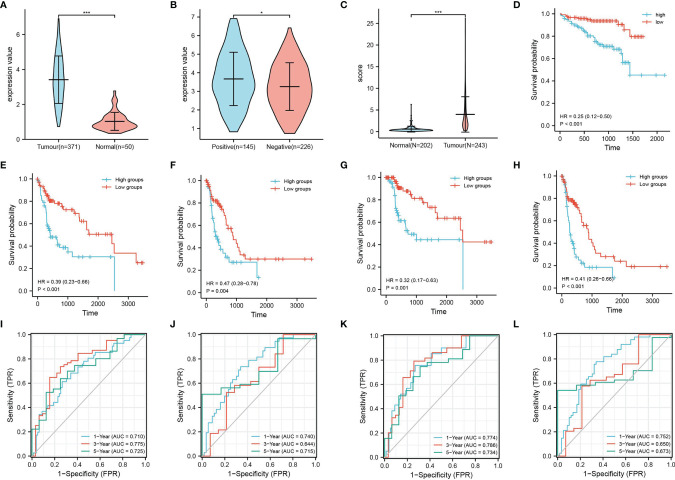
Expression and survival analysis of ASF1B. TCGA database: **(A)** ASF1B expression in liver cancer tissues and normal tissue samples and **(B)** ASF1B expression in HBV-positive and HBV-negative liver cancer samples. ICGC database: **(C)** ASF1B expression in liver cancer tissues and normal tissue samples; **(D)** survival analysis of ASF1B in primary liver cancer; **(E–H)** are the KM survival curves for overall survival (OS), progression-free survival (PFS), disease-free survival (DFS), and disease-specific survival (DSS) of the KM survival curves; and **(I–L)** ROC curves of OS, DFS, DSS, and PFS, respectively. *P < 0.05, ***P < 0.001.

The analysis of samples from the TCGA database revealed that ASF1B was highly expressed in hepatocellular carcinoma tissues, especially HBV-infected hepatocellular carcinoma samples (**Figures 5A, B**). Similarly, samples from the ICGC database yielded the same conclusion, and their high expression was associated with an overall poor prognosis (**Figures 5C, D**). To further understand the prognostic impact of ASF1B, we analyzed the OS, DFS, DSS and PFS based on the TCGA database. The results showed that high ASF1B expression led to a poorer prognosis (**Figures 5E**–**H**). Meanwhile, ROC plots were performed for each prognosis type to predict prognoses at 1, 3, and 5 years, and the diagnostic accuracy of ASF1B was good in all cases (**Figures 5I**–**L**).

### ASF1B Is Associated With the Cell Cycle Signaling Pathway

**Figure 6:**
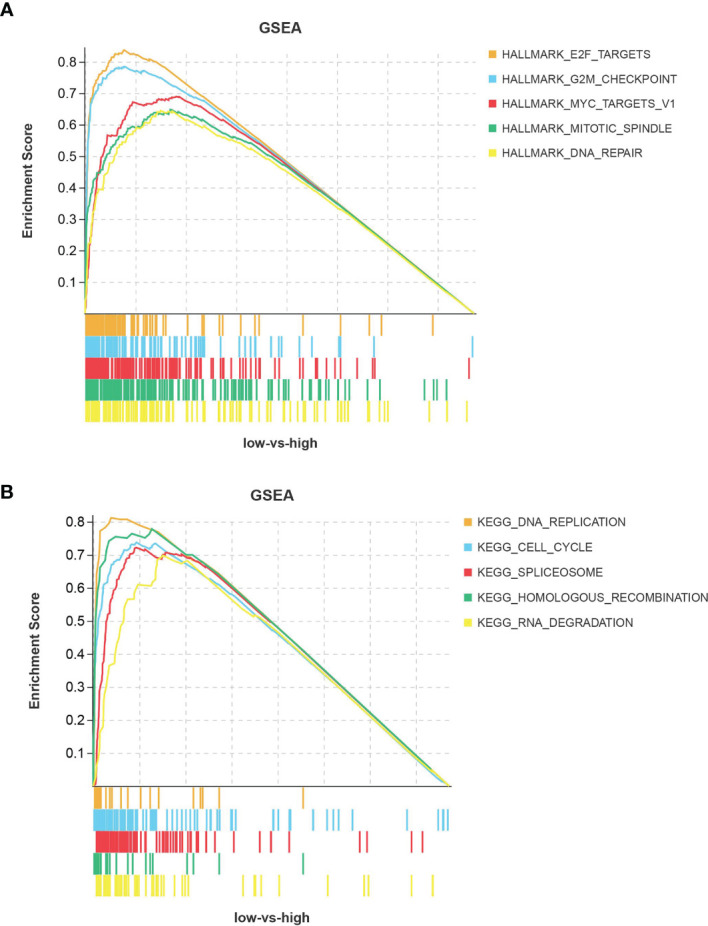
Pathway enrichment analysis of ASF1B. **(A)** Hallmark pathway and **(B)** KEGG pathway.

In HBV-positive liver cancer tissues, ASF1B was mainly enriched in signaling pathways related to cell cycle progression (**Figures 6A, B**). These pathways involve E2F targets, G2M checkpoints, RNA spliceosome assembly, DNA replication, and other pathways, and their activation is an accelerated cell cycle process that facilitates cancer progression. This suggests that ASF1B may promote the progression of HBV-infected hepatocellular carcinoma by affecting cell cycle-related signaling pathways.

## Discussion

With advances in technology and clinical management, there have been remarkable achievements in our understanding of the pathogenesis of liver cancer and in advances in treatment modalities. However, liver cancer’s high morbidity and mortality rates are still of great concern. It is estimated that approximately 240 million people worldwide have been infected with HBV (15). Of these, 70–90% of HBV-infected patients develop hepatocellular carcinoma (16). A high HBV viral load is also an important risk factor for the recurrence of infection after surgery in patients with advanced hepatocellular carcinoma (17). We obtained 850 DEGs by grouping hepatocellular carcinoma patients according to whether they were infected with HBV, and these DEGs were mostly associated with cell differentiation and tissue development. Based on the modeling of prognostic characteristics, we grouped HBV-infected liver cancer samples based on risk scores. The high-risk group in the model was associated with a poor prognosis, and the model had good prognostic predictive power (**Figure 3**). The model included seven pivotal genes associated with prognostic risk and a prognostic independent factor for hepatocellular carcinoma. ASF1B was derived by univariate and multifactorial Cox regression analyses.

Cancer development and progression are associated with the dysregulation of chromatin regulators, including histone variant proteins and histone chaperone proteins (18). ASF1B is an important member of the H3/H4 family of histone chaperone proteins and is mainly involved in cell proliferation (19). ASF1B has a role in regulating the nucleosome structure of chromatin, and its cooperation with chromatin assembly factor 1 (CAF-1) promotes replication-dependent chromatin assembly (20). ASF1B has been identified early in the disease progression of breast cancer. Corpet A et al. noted that overexpression of ASF1B correlated with clinical data and disease outcomes in breast cancer, meaning that ASF1B has diagnostic and prognostic value (21). In recent years, the role of ASF1B in the progression of other cancers has been explored; high ASF1B expression has pointed to a poor prognosis for lung adenocarcinoma and has been associated with advanced tumor stage and tumor progression (22). The expression of ASF1B can also promote multiple myeloma progression (23). In addition, ASF1B expression has been associated with cancer-related pathways. In prostate cancer, ASF1B promotes cancer progression by affecting the PI3K/AKT signaling pathway (24). In cervical cancer, ASF1B promotes cell invasion and affects prognosis by activating the Wnt/β-Catenin signaling pathway (25). However, its role in HBV-infected hepatocellular carcinoma has not been effectively validated. In this study, we analyzed the prognostic significance of ASF1B expression on OS, DFS, DSS, and PFS in HBV-infected hepatocellular carcinoma patients. The results showed that high ASF1B expression led to poorer overall survival and the potential to shorten the interval for tumor progression, tumor recurrence, and death. GSEA showed that high ASF1B expression exhibited great significance regarding pathways associated with cell cycle progression.

Cancer is essentially a group of diseases with persistent excessive cell division tightly regulated by cell cycle control mechanisms (26). The G1 and S phases are two very important periods in the cell cycle, and their changes affect cell proliferation (27). It has been demonstrated that ASF1B is a G1- and S-phase regulator, and the knockdown of ASF1B expression induces G1-phase cell cycle arrest (24). Interestingly, HBV can dysregulate cellular signaling pathways, such as Wnt/FZD/β-catenin, PI3K/Akt/mTOR, and Ras/Raf/MAPK, associated with liver cancer development (28). In this study, we found that the high expression of ASF1B in HBV-infected hepatocellular carcinoma patients was mostly associated with cell cycle-related pathways. However, we mostly focused on functions such as DNA replication, mitosis, etc. ASF1B was previously identified in human islet β-cell replication, and it significantly increased islet β-cell proliferation (29). In addition, ASF1B overexpression promotes melanoma cell growth and adhesion and inhibits apoptosis (30). Furthermore, ASF1B has been shown to promote the proliferation and invasion of clear cell renal cell carcinoma and gastric cancer cells through the activation of the AKT and Bax/Bcl-2-p53 pathways (31, 32).

In conclusion, the present study is the first to analyze ASF1B based on HBV-infected liver cancer tissues. The results showed that ASF1B was highly expressed in HBV-infected compared to non-HBV-infected hepatocellular carcinoma tissues. An association between high ASF1B expression and clinical prognosis was also revealed. Our study showed that ASF1B is an independent prognostic factor in HBV-infected hepatocellular carcinoma and that its high expression leads to a poor prognosis in multiple survival types. In addition, GSEA showed that ASF1B is associated with cell cycle pathways in the progression of HBV-infected hepatocellular carcinoma. These studies contribute to the understanding of the protumor role of ASF1B, but its specific mechanisms require further validation.

## Data Availability Statement

The original contributions presented in the study are included in the article/supplementary material. Further inquiries can be directed to the corresponding authors.

## Author Contributions

All authors listed have made a substantial, direct, and intellectual contribution to the work, and approved it for publication.

## Funding

This work was supported by 2015 Jingzhou Science and Technology Plan (Medical and Health) under grant 2015-04 and Central Funds Guiding the Local Science and Technology Development of Hubei Province under grant 2019ZYYD066.

## Conflict of Interest

The authors declare that the research was conducted in the absence of any commercial or financial relationships that could be construed as a potential conflict of interest.

## Publisher’s Note

All claims expressed in this article are solely those of the authors and do not necessarily represent those of their affiliated organizations, or those of the publisher, the editors and the reviewers. Any product that may be evaluated in this article, or claim that may be made by its manufacturer, is not guaranteed or endorsed by the publisher.
